# Cardiovascular autonomic dysfunction is linked with arterial stiffness across glucose metabolism: the Maastricht study

**DOI:** 10.1136/bmjdrc-2025-004995

**Published:** 2026-03-06

**Authors:** Jonas R. Schaarup, Lasse Bjerg, Christian Stevns Hansen, Signe Toft Andersen, Marleen MJ van Greevenbroek, Miranda T Schram, Bastiaan E De Galan, Coen Stehouwer, Daniel R Witte

**Affiliations:** 1 Department of Public Health, Aarhus University, Aarhus, Denmark; 2 Steno Diabetes Center Aarhus, Aarhus, Denmark; 3 Steno Diabetes Center Copenhagen, Herlev, Denmark; 4 Department of Medicine, Gødstrup Hospital, Herning, Denmark; 5 Internal Medicine, Maastricht University, Maastricht, The Netherlands; 6 Department of Medicine, Maastricht University Medical Center (MUMC+), Maastricht, The Netherlands; 7 Department of Internal Medicine 463, Section Diabetes, Radboud University Nijmegen Medical Center, Nijmegen, The Netherlands; 8 Internal Medicine/Endocrinology, Maastricht University Medical Centre+, Maastricht, The Netherlands; 9 Clinical and Experimental Endocrinology, KU Leuven, Leuven, Belgium

**Keywords:** arteriosclerosis, diabetes mellitus, type 2, population health, autonomic nervous system

## Abstract

**Objective:**

To ascertain the cross-sectional association between cardiovascular autonomic dysfunction and arterial stiffness across glucose metabolism status.

**Research design and methods:**

We performed a cross-sectional analysis of participants of the Maastricht study. Cardiovascular autonomic function was based on heart rate variability (HRV) indices from 24-hour ECG recordings and summarized in z-scores for time and frequency domains. Aortic and carotid stiffness were assessed by carotid-femoral pulse wave velocity (PWV) and carotid artery distensibility (CD), respectively. We used multiple linear regression to study the associations and adjusted for demographic and lifestyle factors and a range of cardiovascular risk factors. We tested for effect modification of the associations by glucose metabolism status.

**Results:**

PWV and CD measures were available in 3673 and 1802 participants, respectively (median (25th; 75th percentile) age: 60 years (53; 66), 51% women, 20% type 2 diabetes by design. Participants with lower HRV had higher aortic stiffness. Per SD lower time-domain and frequency-domain HRV z-scores were associated with 2.8% (95% CI 2.1% to 3.4%) and 2.8% (95% CI 2.1% to 3.5%) higher PWV, respectively. Similar trends were observed for carotid stiffness, with 3.2% (95% CI 1.4% to 5.0%) and 3.1% (95% CI 1.2% to 5.0%) lower CD per SD lower time-domain and frequency-domain HRV, respectively. The magnitude of these associations was higher in groups with prediabetes and type 2 diabetes compared with those with normal glucose metabolism, with evidence of effect modification by glucose metabolism status (p value for interaction: <0.01 for prediabetes and <0.05 to <0.10 for type 2 diabetes, both compared with normal glucose metabolism).

**Conclusion:**

Cardiovascular autonomic dysfunction is associated with higher aortic and carotid stiffness, especially in people with dysglycemia.

**WHAT IS ALREADY KNOWN ON THIS TOPIC**

- The mechanisms explaining the link between cardiovascular autonomic dysfunction and cardiovascular disease are not well understood but may involve vascular stiffness.
- Investigating their interplay across glucose metabolism statuses could provide insights into how vascular changes unfold as people progress toward diabetes.

**WHAT THIS STUDY ADDS**

- In this study, we aimed to ascertain the association between autonomic dysfunction, measured by 24-hour heart rate variability (HRV), and arterial stiffness and whether the association is modified by different glucose levels.
- We found that lower 24-hour HRV was associated with both aortic and carotid stiffness.
- The association was as strong in prediabetes as in those with type 2 diabetes.

**HOW THIS STUDY MIGHT AFFECT RESEARCH, PRACTICE OR POLICY**

- Autonomic dysfunction may elevate cardiovascular risk by influencing vascular stiffness, particularly in prediabetes and diabetes.
- With wearable devices becoming more accessible, we have shown how 24-hour HRV can serve as an indicator of cardiovascular disease risk.

## Background

 Arterial stiffness is affected by the cumulative effects of cardiometabolic risk factors and is an established surrogate end point for atherosclerotic cardiovascular disease (CVD) events, including myocardial infarction and stroke.^1 2^ Improvement of targeted CVD prevention and treatment in people with diabetes and prediabetes requires a deeper understanding of the interplay between early stages of CVD and diabetes complications.^3 4^ Cardiovascular autonomic dysfunction (autonomic dysfunction), expressed by a reduction in heart rate variability (HRV), is an established risk indicator for CVD that can be easily monitored by wearables, such as smartwatches.^5 6^ However, the mechanisms that explain the link between autonomic dysfunction and CVD remain unclear. Arterial stiffness reflects structural changes in the arterial wall as, with aging, the elastin fibers gradually are substituted with collagen fibers in the media layer of the large arteries.^7^

Cardiovascular autonomic function can be estimated by HRV indices. The variation between the distance of successive normal interbeat intervals (IBI) in milliseconds forms the basic observation underlying all HRV indices. It provides a time-domain or frequency-domain estimate of the balance between the sympathetic and parasympathetic tone influencing the sinoatrial node.^8^ Lower HRV indices characterize autonomic dysfunction, which may initially be reflected by sympathetic overactivity and reduced vagal activity.^9^

Autonomic dysfunction is regarded as a late complication of diabetes, and its association with arterial stiffness is well established.^10 11^ However, these findings are primarily based on ECG-derived measurements obtained during short-term recordings under standardized resting conditions.^10 11^ Extended recordings of HRV covering the circadian rhythms of sympathetic and parasympathetic activity may give insight into the role of lower-frequency sources of variability, that is, very low frequency and ultra-low frequency.^8 12^ Lower 24-hour HRV reflects poorer adaptation in cardiac and vascular response to internal and external stimuli throughout the circadian rhythm.^12 13^ Yet, the extent to which long-term HRV components influence arterial stiffness remains unclear. Moreover, the Whitehall II study showed a longitudinal link between short-term HRV and aortic stiffness in the general population, implying that the association can be observed without the presence of diabetes.^14^ Understanding how dysglycemia modifies the relationship between autonomic dysfunction and arterial stiffness may clarify the extent to which increased CVD risk in individuals with prediabetes and type 2 diabetes is driven by the progression of autonomic dysfunction.

In studies of autonomic dysfunction and arterial stiffness, most have measured arterial stiffness based solely on aortic stiffness.^11^ A separate investigation of both aortic stiffness and carotid stiffness reflects different components of the arterial tree structure that are differently associated with types of CVD events.^15 16^ This etiological cross-sectional study aimed to ascertain the association between cardiovascular autonomic function, measured by 24-hour HRV, and arterial stiffness across glucose metabolism status. We hypothesized that autonomic dysfunction, expressed by lower HRV, is associated with higher levels of aortic and carotid stiffness and that the association is more pronounced in people with more advanced dysglycemia.

## Research design and methods

### Data collection

The exact description of the Maastricht study is referenced from previous publications^17^: We used data from the Maastricht study, an observational prospective population-based cohort study. The rationale and methodology have been described previously. In brief, the study focuses on the etiology, pathophysiology, complications, and comorbidities of type 2 diabetes mellitus (T2DM) and is characterized by an extensive phenotyping approach. Eligible for participation were all individuals aged between 40 and 75 years and living in the southern part of the Netherlands. Participants were recruited through mass media campaigns and from the municipal registries and the regional Diabetes Patient Registry via mailings. Recruitment was stratified according to known T2DM status, with an oversampling of individuals with T2DM, for reasons of efficiency.

The examinations of each participant were performed within a time window of 3 months. We examined participants who had both HRV and measurements of aortic and carotid stiffness within a 3-month window around the baseline examination round of the Maastricht study.^17^

The present study includes cross-sectional data from the first 7449 participants, who completed the baseline survey between November 2010 and December 2020 and had measures of arterial stiffness assessed, processed, and cleaned. We excluded participants who self-reported prior CVD events, as their pathophysiology and consequent treatment could influence both arterial structural changes and impairment of autonomic balance. We also excluded participants with other types of diabetes than type 2 diabetes, as we investigated the effect modification by glucose metabolism status.

### Exposure

All ECG recordings were obtained using a 12-lead Holter system (Fysiologic ECG Services, Amsterdam, the Netherlands) over 24 hours. The procedure for data collection has previously been reported.^13^ During the recording period, participants were instructed to keep their regular daily activities but were asked to refrain from showering. The recorded ECG data were then processed using proprietary Holter Analysis Software at Fysiologic ECG Services. Non-sinus cardiac cycles, that is, artifacts and premature/ectopic beats, were excluded. This process was subsequently validated through manual inspection. Following the exclusion of non-sinus cardiac cycles, the minimum required recording duration for ECG analysis was set at 18 hours. The software from Fysiologic ECG Services provided the IBI in milliseconds between individual R waves of sinus beats. HRV indices were computed using the publicly available GNU Octave software,^18^ including the time and frequency domain measures established by the Task Force recommendation on HRV.^8^ Time-domain HRV indices were calculated, including the SD of all normal-to-normal (NN) intervals (SDNN, in ms), the SD of the averages of NN intervals in 5 min segments throughout the recording (SDANN, in ms), the square root of the mean of the sum of squares of differences between adjacent NN intervals (RMSSD, in ms), the mean of the SDs of all NN intervals for all 5 min segments (SDNN index, in ms), and the NN50 count divided by the total number of all NN intervals (pNN50, percentage). Frequency-domain HRV measures were determined using the Fast Fourier Transform based on spectral segment for the whole recording cycle. In the frequency-domain HRV, ms² measures the power or energy of the HRV signal within predefined frequency bands. These included the variance of all NN intervals ≤0.4 Hz, total power (TP, in ms^2^), power in the ultra-low-frequency range (ULF, in ms^2^ ≤0.003 Hz), power in the very-low-frequency range (VLF, in ms^2^; 0.003–0.04 Hz), power in the low-frequency range (LF, in ms^2^; 0.04–0.15 Hz), and power in the high-frequency range (HF, in ms^2^; 0.15–0.4 Hz). We removed outliers in time-domain and frequency-domain HRV indices (see description in the online supplemental material). We standardized HRV indices by their mean and SD to make indices comparable and calculated composite z-scores for time and frequency-domain HRV indices, respectively. The time-domain z-score included: SDNN, SDANN, RMSSD, SDNN index, and pNN50, and the frequency-domain z-score included: TP, HF, LF, VLF, and ULF. Prior evidence shows that this selection of indices covers most of the underlying sources of variance determined by calculations of IBI.^8^ We also included mean IBI for comparison with the HRV indices.

### Outcome

Aortic and carotid stiffness were included as measures for arterial stiffness. The procedure for arterial measurements has been previously documented.^19^ Aortic stiffness was determined by carotid-femoral pulse wave velocity (PWV) and was assessed using applanation tonometry (SphygmoCor, Atcor Medical, Sydney, Australia). We included the median value from at least three consecutive PWV recordings in our analyses.

Carotid stiffness was determined by the carotid artery distensibility coefficient (CD). Ultrasound examinations of the left common carotid artery using a 7.5 MHz linear probe-equipped ultrasound scanner (MyLab 70, Esaote Europe, Maastricht, the Netherlands) were conducted to evaluate local carotid distension. Local carotid stiffness was quantified by computing the CD, using the following equation:


$$CD=\frac{\left(2\times \Delta D\times IAD+\Delta D^{2}\right)}{\left(braPP\times IAD^{2}\right)\;\left(10\;3\;kPa-1\right)},$$

where ΔD represents distension, and braPP signifies brachial pulse pressure. Alongside the vascular assessments, mean heart rate and mean arterial pressure (MAP) were monitored at 5 min intervals using an oscillometer device (Accutorr Plus, Datascope, Montvale, New Jersey, USA).

### Covariates

Lifestyle factors of smoking (never, former (quit >6 months ago), former (quit <6 months ago), current), physical activity: total (hours/week) and moderate to vigorous exercise (hours/week), and alcohol consumption (average units per week), as well as CVD disease history, and antihypertensive, glucose-lowering, and lipid-lowering medication use, were reported through a self-reported questionnaire. Hemoglobin A1c (HbA1c), fasting plasma glucose (FPG), triglycerides, and total, high-density lipoprotein (HDL), and low-density lipoprotein (LDL) cholesterol levels were measured in blood samples. Anthropometric measures of body mass index (BMI) and waist circumference, as well as systolic and diastolic blood pressure, were measured at the study site.^17^ We used WHO 2006 criteria for categorizing glucose metabolism status into normal glucose metabolism, prediabetes (impaired fasting glucose and impaired glucose tolerance), and type 2 diabetes, based on a 2-hour 75 g oral glucose tolerance test and/or the use of glucose-lowering medication.^20^ HbA1c was not used as a criterion for type 2 diabetes or prediabetes.

### Statistical analysis

We describe population characteristics by the distribution (median, 25th and 75th percentile) for continuous variables and frequencies (numbers, percentage) for categorical variables.

We performed multiple linear regression with HRV indices as exposure for the outcome of arterial stiffness. We included the glucose metabolism status (normal glucose metabolism, prediabetes, and type 2 diabetes) to account for the oversampling of individuals with known type 2 diabetes. We further adjusted for MAP to account for potential instrumental bias, ensuring that elevated MAP during the measurement of arterial stiffness does not falsely indicate greater stiffness.^21^ Model 1 was adjusted for age, sex, education, MAP, and glucose metabolism status. In model 2, we further adjusted for self-reported total physical activity (hours/week), smoking behavior, alcohol use, BMI, HbA1c, triglycerides, total-to-HDL cholesterol ratio, lipid-modifying and antihypertensive medication. Blood pressure measures other than MAP are considered a collider as they are affected by autonomic dysfunction and arterial stiffness and thus were not included in the model.^22^ To obtain normally distributed residuals, we log-transformed measures for arterial stiffness (PWV and CD) and back-transformed the model estimates into a percentage scale. We further tested for effect modification by sex and diabetes status by including them as multiplicative interaction terms in separate models. A significant interaction was determined by a p value <0.05. We also carried out a subsidiary analysis to investigate possible gradual stratified modification by higher glucose levels, using 20th percentiles of either FPG or HbA1c after excluding people using glucose-lowering medication. To test the robustness of our analysis, we performed a sensitivity analysis first excluding individuals with antihypertensive treatment and subsequently people with type 2 diabetes. In the effect modification analysis by diabetes status, we performed an additional analysis excluding people using beta-blockers. We performed a complete case analysis, using the statistical program R (V.4.3.2).^23^

## Results

### Descriptive

**Figure 1:**
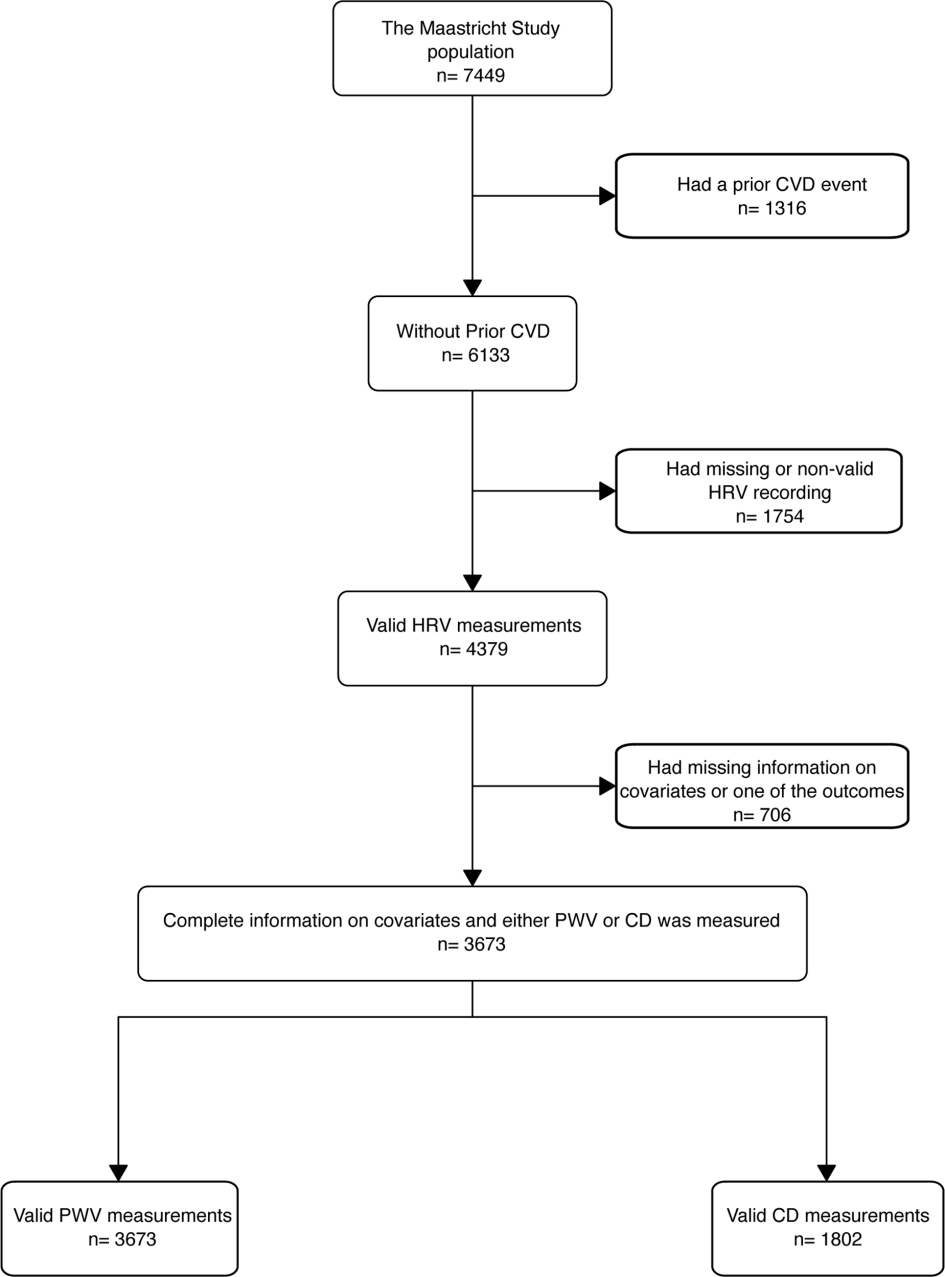
Study flow chart. CD, carotid artery distensibility; CVD, cardiovascular disease; HRV, heart rate variability; PWV, pulse wave velocity.

Of the whole study population with available measures of HRV without prior CVD events and other types of diabetes, 3673 had PWV and 1802 had CD measured (figure 1). Details of excluded participants are provided in the online supplemental material (tables S1 and S2). Fifty-one per cent were women and participants had a median (25th; 75th percentile) age of 60 (53; 66) years, and 2387 (65%), 537 (15%), and 747 (20%) had normal glucose metabolism, prediabetes, and type 2 diabetes, respectively (table 1). The population with type 2 diabetes more frequently used lipid-lowering and antihypertensive medication compared with the populations with prediabetes or normal glucose metabolism (online supplemental table 3S). The median SDNN (HRV) was 133 ms (110; 158). The median PWV (aortic stiffness) was 8.40 (7.44; 9.76) m/s and CD (carotid stiffness) was 14.2 (11.0; 17.8) 10^−3^/kPa.

**Table 1:** Study population characteristics

	N=3673
Women	1884 (51%)
Age (years)	60 (53, 66)
White ethnicity	3633 (99%)
Education	
Low (no education, (un)completed primary education, or lower vocational education)	1094 (30%)
Middle (intermediate vocational education or higher secondary education)	1050 (29%)
High (higher vocational education or university education)	1529 (42%)
Alcohol total (g/day)	9 (2, 19)
Smoking status	
Never	1417 (39%)
Former (quit >6 months ago)	1733 (47%)
Former (quit <6 months ago)	62 (1.7%)
Current	461 (13%)
Total physical activity (hours/week)	13 (8, 19)
BMI (kg/m^2^)	26.0 (23.6, 28.8)
HbA1c (%)	5.54 (5.26, 5.90)
Fasting plasma glucose (mmol/L)	5.40 (4.90, 6.00)
LDL (mmol/L)	3.10 (2.40, 3.80)
HDL (mmol/L)	1.50 (1.20, 1.90)
Total cholesterol (mmol/L)	5.30 (4.60, 6.10)
Triglycerides (mmol/L)	1.18 (0.87, 1.65)
Glucose metabolism status	
Normal glucose metabolism	2389 (65%)
Prediabetes	538 (15%)
Type 2 diabetes	746 (20%)
Duration of type 2 diabetes (only for diagnosed participants)	3 (0, 9)
Mean IBI (ms)	828 (765, 904)
SDNN (ms)	133 (110, 158)
RMSSD (ms)	25 (20, 34)
SDANN (ms)	119 (97, 143)
TP (ms^2^)	11 566 (7991, 16 394)
ULF (ms^2^)	9788 (6655, 14 183)
VLF (ms^2^)	1105 (736, 1571)
LF (ms^2^)	364 (222, 593)
HF (ms^2^)	84 (50, 149)
Systolic blood pressure (mm Hg)	126 (116, 136)
Diastolic blood pressure (mm Hg)	76 (71, 81)
Mean arterial pressure (mm Hg)	96 (89, 103)
Carotid artery distensibility (10^–3^/kPa)	14.2 (11.0, 17.8)
Carotid-femoral pulse wave velocity (m/s)	8.40 (7.44, 9.76)
Diagnosed hypertension	1740 (47%)
Glucose-lowering medication	519 (14%)
Antihypertensive medication	1108 (30%)
Beta-blockers	421 (11%)
Diuretic aldosterone	15 (0.4%)
Diuretics	470 (13%)
Lipid-lowering medication	905 (25%)

Data are shown as n (%) or median (IQR).

BMI, body mass index; HbA1c, hemoglobin A1c; HDL, high-density lipoprotein; HF, high frequency; IBI, interbeat interval; LDL, low-density lipoprotein; LF, low frequency; RMSSD, the square root of the mean of the sum of squares of differences between adjacent NN intervals; SDANN, the SD of the averages of NN intervals in 5 min segments throughout the recording; SDNN, the SD of normal-to-normal R-R intervals; TP, total power; ULF, ultra-low frequency; VLF, very low frequency.

### Heart rate variability and aortic stiffness

**Figure 2:**
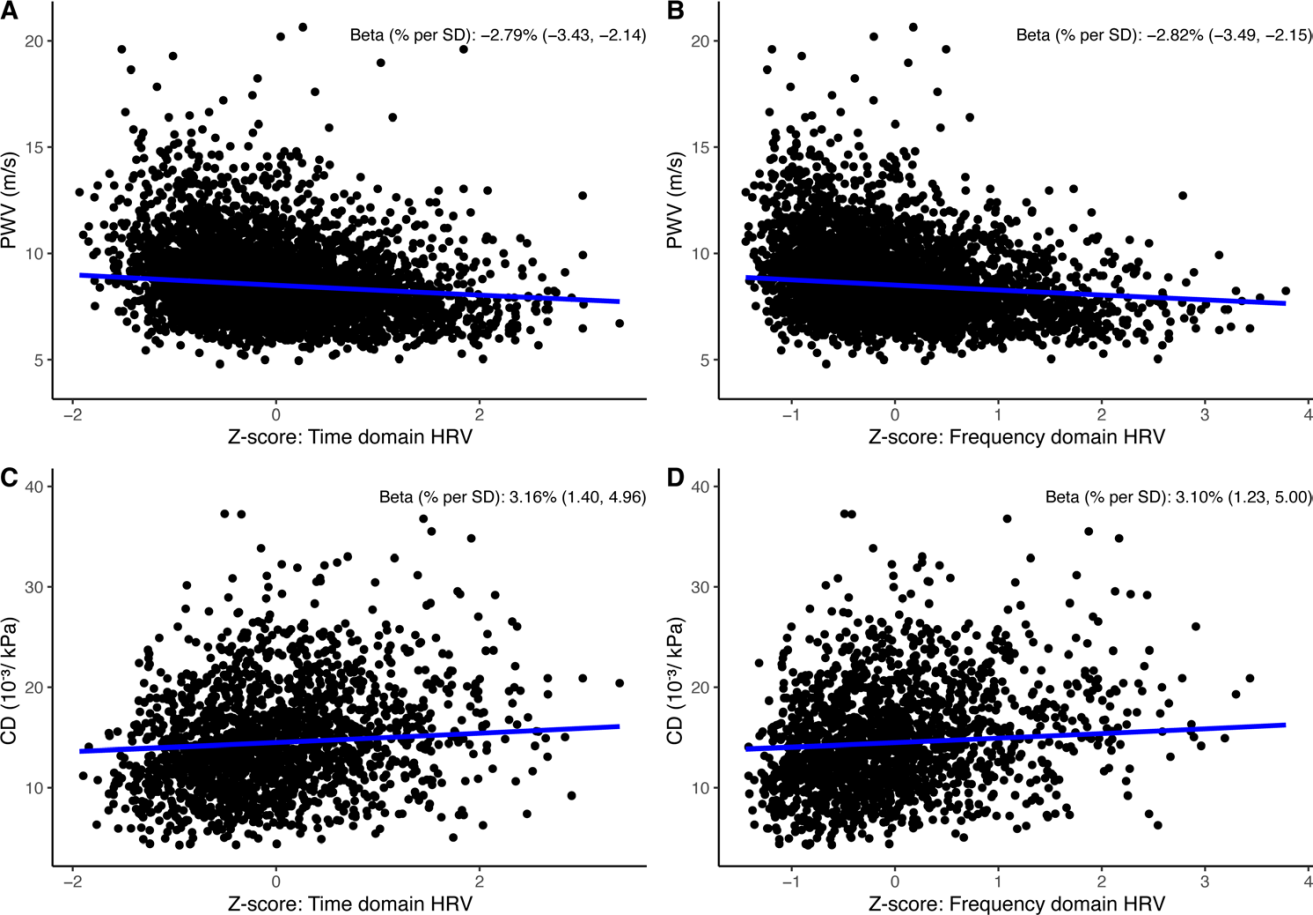
Linear relationship between 24-hour HRV and aortic and carotid stiffness. (**A**) Percentage PWV per SD in time-domain composite z-score. (**B**) Percentage PWV per SD in frequency-domain composite z-score. (**C**) Percentage higher CD per SD in time-domain composite z-score. (**D**) Percentage CD per SD in frequency-domain composite z-score. All regression lines were adjusted for sex, age, educational level, diabetes status, and mean arterial pressure. CD, carotid artery distensibility; HRV, heart rate variability; PWD, pulse wave velocity.

**Figure 3:**
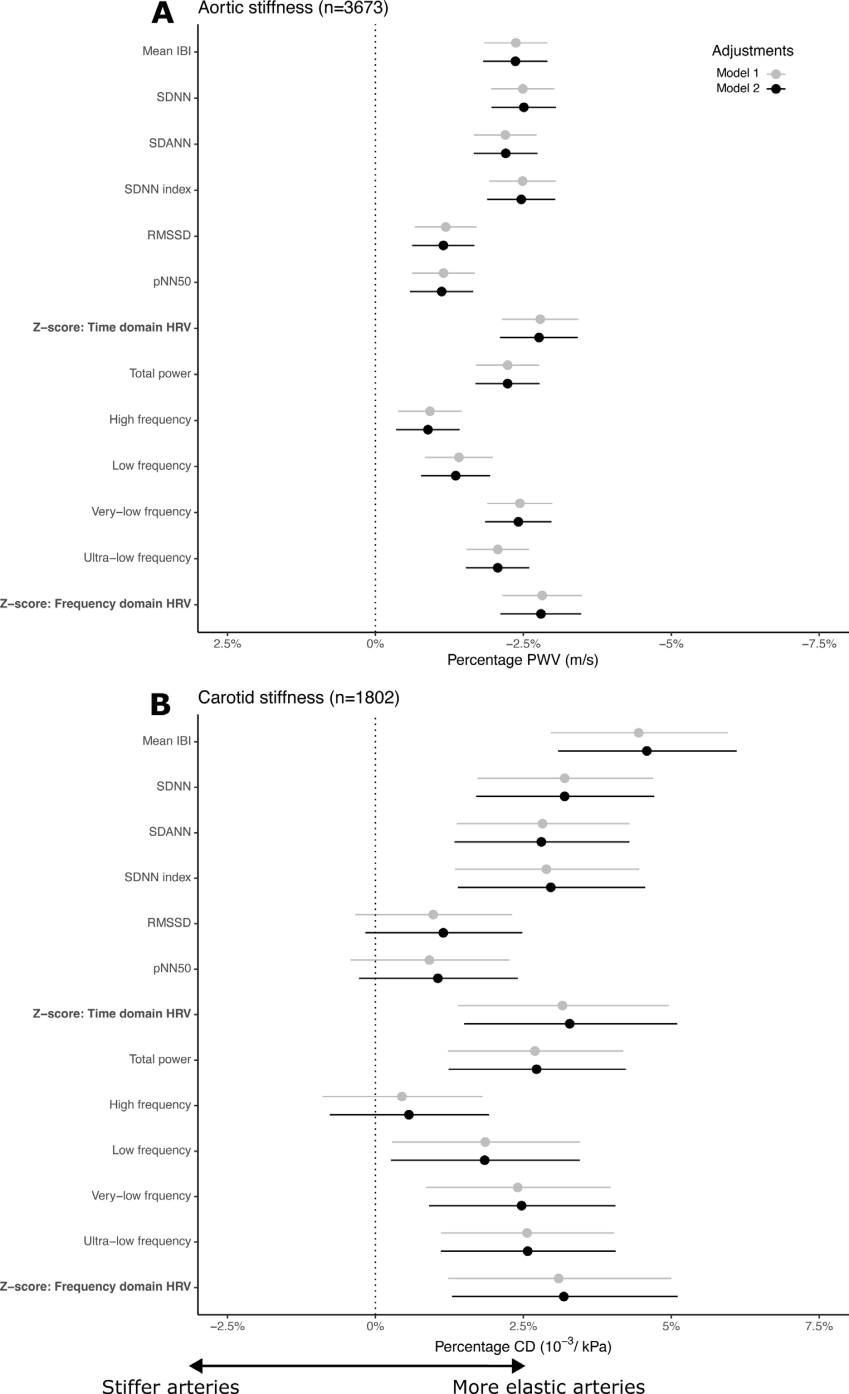
Association between 24-hour HRV and arterial stiffness. Percentage PWV (**A**) or CD (**B**) per SD increase in heart rate variability index and heart period intervals. Model 1: adjusted for sex, age, educational status, diabetes status, and mean arterial pressure. Model 2: model 1+physical activity, smoking behavior, alcohol use, body mass index, hemoglobin A1c, triglycerides, total-to-high-density lipoprotein cholesterol ratio, lipid-modifying and antihypertensive medication. Z-score: frequency-domain HRV. CD, carotid artery distensibility; HRV, heart rate variability; IBI, interbeat interval; pNN50, the NN50 count divided by the total number of all NN intervals; PWV, pulse wave velocity; RMSSD, the square root of the mean of the sum of squares of differences between adjacent NN intervals; SDNN, the SD of normal-to-normal R-R intervals; SDANN, the SD of the averages of NN intervals in 5 min segments throughout the recording.

In model 1, for each SD lower HRV time-domain z-score, PWV was 2.78% (95% CI 2.13 to 3.42) higher. For each SD lower HRV frequency-domain z-score, PWV was 2.82% (95% CI 2.14 to 3.49) higher (figure 2A,B). The strongest associations were seen in SDNN and SDANN for the time domain and in total power, VLF, and ULF for the frequency domain (figure 3A and online supplemental table S4 and S12). Associations did not change materially on adjustment for the confounders in model 2. The sensitivity analyses showed that excluding participants using antihypertensive medication did not materially change the estimates(online supplemental table S10). No interaction was observed by sex (online supplemental table S8).

### Heart rate variability and carotid stiffness

In model 1, for each SD lower HRV time-domain z-score, CD was 3.17% (95% CI 1.41 to 4.96) lower. For each SD lower HRV frequency-domain z-score, CD was 3.12% (95% CI 1.24 to 5.01) lower (figure 2C,D). The strongest associations were seen in SDNN and SDANN for time-domain indices and in total power, VLF, and ULF for the frequency domain (figure 3A,B and online supplemental table S5 and S13)). Associations did not change materially on adjustment for the confounders in model 2. Except for the HRV index VLF, the sensitivity analyses showed that excluding participants using antihypertensive medication did not materially change the estimates (online supplemental table S11). No interaction was observed by sex (online supplemental table S9).

### Effect modification by glucose metabolism status

**Figure 4:**
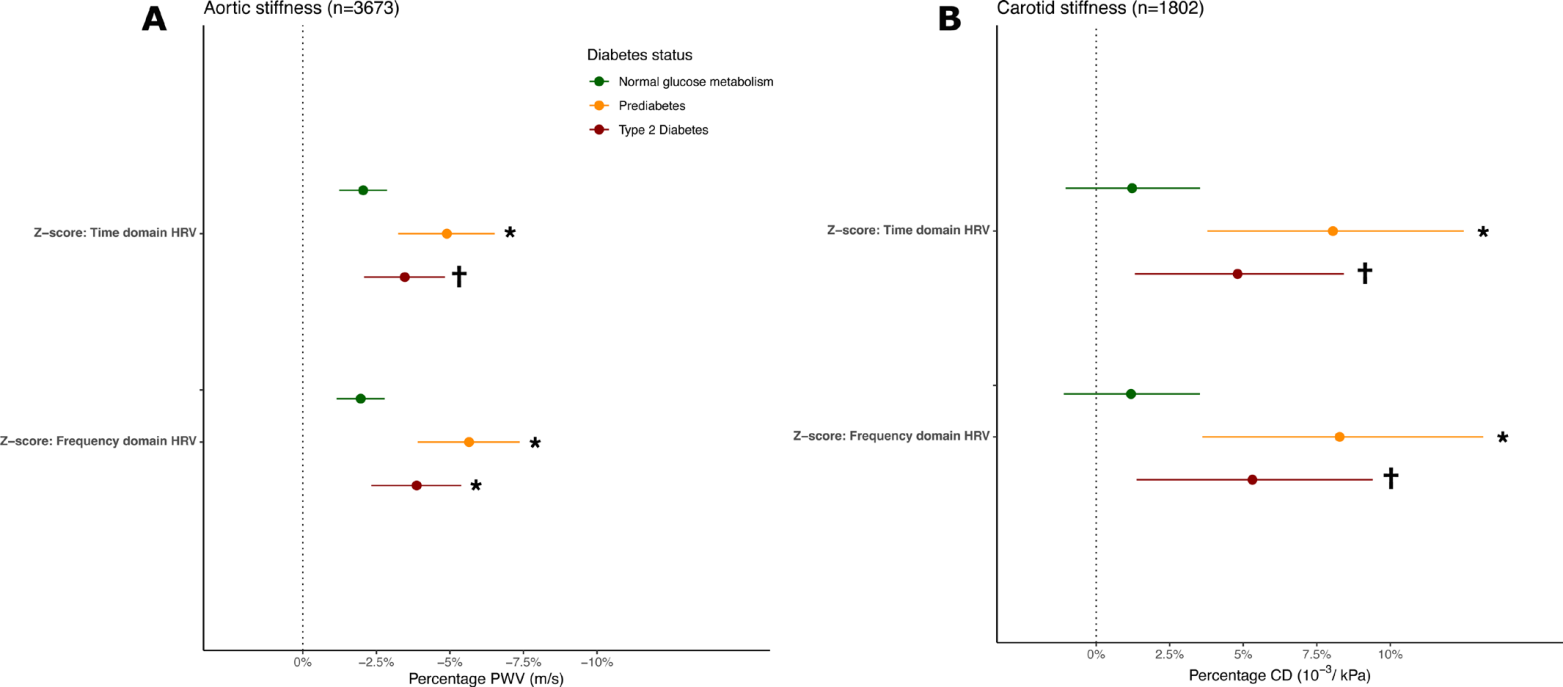
Association between 24-hour HRV and arterial stiffness modified by diabetes status. (**A**) Percentage PWV per SD in time-domain and frequency-domain composite z-score by diabetes status. (**B**) Percentage CD per SD in time-domain and frequency-domain composite z-score by diabetes status. Estimates are adjusted for sex, age, educational status, mean arterial pressure, physical activity, smoking behavior, alcohol use, body mass index, hemoglobin A1c, triglycerides, total-to-high-density lipoprotein cholesterol ratio, lipid-modifying and antihypertensive medication. Normal glucose metabolism was defined as the reference group. *Interaction term p value <0.05. †Interaction term p value <0.10. CD, carotid artery distensibility; HRV, heart rate variability; PWV, pulse wave velocity.

The association between HRV and measures of arterial stiffness was stronger in people with prediabetes and type 2 diabetes than in those with normal glucose metabolism (figure 4A,B). Indeed, we observed statistically significant interactions when comparing prediabetes and normal glucose metabolism, whereas the interaction was only significant for type 2 diabetes in the association between HRV frequency-domain z-score and PWV. Excluding people using beta-blockers raised the estimates for the type 2 diabetes group in the analysis with PWV as outcome but not in CD (online supplemental figure S3). Effect modification estimates for each HRV index are presented in the online supplemental tables S6 and S7. When we excluded people using glucose-lowering medication and analyzed the stratified modification by quintiles of glycemia, we found stronger associations between the frequency-domain and time-domain z-score and PWV and CD in higher percentiles of FPG and HbA1c (online supplemental figures S1 and S2).

## Discussion

In this study, we showed that cardiovascular autonomic dysfunction, determined by lower long-term HRV, was associated with both aortic and carotid stiffness among middle-aged to elderly adults. This association was present regardless of glucose metabolism status, but appeared stronger among individuals with prediabetes or type 2 diabetes.

Several studies found lower HRV indices to be associated with aortic stiffness among people with either type 1 or type 2 diabetes.^11 14^ Our study extends these findings by showing that the associations are already present in people without diabetes, although to a lesser degree than in people with prediabetes or diabetes.

The magnitude of the observed associations was modest but relevant when compared with equivalent associations of age with arterial stiffness. One SD lower HRV was equivalent to the effect of 2.7 additional years on PWV and to 1.6 years for CD. Earlier studies have demonstrated that short-term HRV is linked with both coronary heart disease and stroke,^24 25^ but these associations are less clear for long-term HRV. Our data extend these findings, showing that the association between long-term HRV and surrogate markers of CVD, such as aortic stiffness and carotid stiffness, supports the notion that long-term HRV links with both myocardial infarction and stroke.^15 16^

We accounted for the oversampling of people with type 2 diabetes by adjusting for diabetes status and correcting for the instrument bias in stiffness measures, caused by higher pulse pressure during measurement of PWV and CD, by adjusting for MAP. Adjustment for lifestyle habits and cardiovascular risk factors did not materially change the estimates, suggesting most of the measurable confounding was captured by diabetes status and MAP. In our sensitivity analysis, without participants on antihypertensive treatment, the estimates did not materially change, thus we focused on models for the entire study population and adjusted for medication in the full model.

Our findings indicate that, beyond autonomic dysfunction assessed by short-term HRV under resting conditions, dysfunction defined by long-term HRV is also associated with arterial stiffness. This suggests that a blunted ability of the autonomic nervous system to modulate IBI appropriately during rest and activity across the diurnal cycle may contribute to structural vascular changes. The association is primarily reflected in HRV indices capturing global IBI variation (SDNN, SDANN, and SDNN index) and lower-frequency bands (LF, VLF, and ULF). We showed that shorter mean IBI was associated with both aortic and carotid stiffness, emphasizing a potential mediating role for higher heart rate in autonomic dysfunction. Sympathetic predominance may result in a higher heart rate and hence lead to higher shear stress on the arterial wall.^26^ The association might also be driven by direct sympathetic effects on arteries, caused by increased levels of norepinephrine and reduced clearance.^27 28^ The level of HRV may depend on heart rate. We did not include adjustment of heart rate in the model as we believe it violates the principles of multicollinearity. Moreover, as a higher heart rate is determined by increased sympathetic bursts, this supports its role as a mediator on the pathway from autonomic dysfunction to arterial stiffness.^28^

Both cardiovascular autonomic dysfunction and arterial stiffness are likely to be shared consequences of cardiometabolically disturbed environment including dyslipidemia, hyperinsulinemia, and advanced glycation end-products induced by hyperglycemia, oxidative stress, and inflammation.^1329^,^33^ We attempted to mimic an etiological ordering by showing the temporality of glucose metabolism (normal glucose metabolism, prediabetes, and type 2 diabetes) in the relationship between autonomic dysfunction and arterial stiffness. Our results support the notion that hyperglycemia modifies the association between HRV and arterial stiffness, as we found stronger associations in the higher quintiles of both FPG and HbA1c in participants without glucose-lowering medication. Early deterioration of glucose metabolism starts a complex cycle of complications, in this case, autonomic dysfunction that, along with dysglycemia and likely through neuronal damage affecting parasympathetic modulation,^13^ may contribute to vascular dysfunction. Although our data are cross-sectional, the observed effect modification gives a notion that the CVD risks are higher in prediabetes and that improvement of glycemic control could potentially in part modify the contribution of low HRV to arterial stiffness.

Two explanations might clarify why the effect modification did not increase progressively by glycemic status in the present study. First, because of being diagnosed with type 2 diabetes, participants were more likely to receive cardioprotective care, including glucose-lowering, lipid-lowering, and antihypertensive medication, an effect that cannot be accounted for by adjustment. After exclusion of people using beta-blockers, the results partly explained why type 2 diabetes showed a smaller modifying effect compared with prediabetes in the outcome of PWV, but not in the outcome of CD. The second explanation could be due to selection bias, as participants with type 2 diabetes, who participated in the Maastricht study and underwent both long-term ECG recordings and measures of arterial stiffness might be healthier than the background population with type 2 diabetes.

Hyperglycemia is rarely an isolated risk factor among people with prediabetes and type 2 diabetes. Therefore, current type 2 diabetes guidelines focus on multifactorial cardiometabolic management, the effect of which on microvascular and macrovascular complications has been clearly demonstrated.^10 34^ Closer attention to the mechanisms that mediate these effects offers the prospect of new intervention points. Although it is conceivable that multifactorial risk management slows the progression of arterial stiffening partially by modulating autonomic dysfunction, it remains to be proven whether modification of HRV per se contributes causally to reduction of CVD risk. To ascertain this causality, observational studies using Mendelian randomisation would provide a first line of evidence. Furthermore, cardiometabolic trials assessing either lifestyle modification or pharmacological interventions should, if possible, measure HRV to enable a structured mediation analysis.

Our findings help to understand that the progression of autonomic dysfunction plays a role in CVD risk and confirm that prediabetes defines a group with an underappreciated higher risk of macrovascular complications.^4^ Lifestyle and glucose-lowering interventions improve cardiometabolic outcomes in prediabetes but have not yet been shown to effectively prevent CVD or all-cause mortality events.^35^ Autonomic dysfunction may serve as a tool for risk stratification among individuals with prediabetes who have high CVD risk. These individuals may particularly benefit from lifestyle interventions to reduce their CVD risk.^36^ Lastly, our findings show that autonomic dysfunction plays a smaller, but still meaningful, role in CVD risk among people without diabetes.

The strengths of the study are the large sample size with a large subpopulation with type 2 diabetes and that HRV was determined by long-term 24-hour ECG recordings in free-living conditions capturing heartbeats in rest and activity representing valid autonomic assessment in a full-day cycle.^8^ Some limitations merit consideration. First, non-stationary activity (including physical activity, timing of meals and caffeinated drinks, and sleeping patterns) can cause IBI variation, potentially reflecting behavioral influences on HRV rather than cardiovascular autonomic function.^8^ We included self-reported total physical activity to account for habitual physical activity. Future research investigating cardiovascular autonomic nervous responses to specific physical activities may help identify patterns that reflect CVD risk in relation to behavioral exposures under free-living conditions.^37^ Second, the generalizability of our findings is limited to middle-aged to elderly populations of white ethnicity with access to high-quality diabetes care. Finally, our study is based on cross-sectional data and thus, we cannot infer a causal direction. The association between HRV and arterial stiffness might be bidirectional, in that arterial remodeling may also cause changes in autonomic balance.^38 39^ However, prior evidence suggests that autonomic dysfunction is mainly contributing to arterial stiffness rather than the other way around.^14^

Wearable devices have made data collection of physiological measures more accessible in general populations, for example, by smartwatches.^5^ Hence, long-term HRV is becoming more accessible to users and eventually healthcare providers. However, its clinical relevance and role remain to be ascertained before implementation. A 24-hour cycle of long-term HRV measured by wearable devices may serve as an easy and non-invasive tool to silently identify individuals with elevated CVD across all stages of diabetes risk.

In conclusion, lower 24-hour HRV was associated with both higher aortic and carotid stiffness, with stronger associations observed in individuals with worse glucose metabolism status. Cardiovascular autonomic dysfunction may contribute to cardiovascular risk by affecting vascular stiffness. Therefore, cardiovascular autonomic function might be a relevant risk indicator of efforts to prevent trajectories toward CVD mediated through arterial stiffness, even before the onset of diabetes.

## Supplementary material

10.1136/bmjdrc-2025-004995 online supplemental file 1

## Data Availability

Data are available on reasonable request.
